# Neonatal and maternal outcomes of mRNA versus Non-mRNA COVID-19 vaccines in pregnant patients: a systematic review and meta-analysis

**DOI:** 10.61622/rbgo/2024rbgo69

**Published:** 2024-09-18

**Authors:** Juliana Almeida Oliveira, Eloisa Gonçalves da Silva, Ayse Filiz Gokmen Karasu, Anelise Maria Nicolau Silva, Chris Elizabeth Philip

**Affiliations:** 1 Universidade Federal de Minas Gerais Belo Horizonte MG Brazil Universidade Federal de Minas Gerais, Belo Horizonte, MG, Brazil.; 2 Centro Universitário de Jaguariúna Jaguariúna SP Brazil Centro Universitário de Jaguariúna, Jaguariúna, SP, Brazil.; 3 Bezmialem Vakif University Istanbul Turkey Obstetrician and Gynecologist, Bezmialem Vakif University, Istanbul, Turkey.; 4 Escola Bahiana de Medicina e Saúde Pública Salvador BA Brazil Escola Bahiana de Medicina e Saúde Pública, Salvador, BA, Brazil.; 5 Beaumont hospital Dublin Ireland Obstetrician and Gynecologist, Beaumont hospital, Dublin, Ireland.

**Keywords:** mRNA vaccines, Pregnant women, Pregnancy complications, Infant, newborn, COVID-19 vaccines, COVID-19, SARS-CoV-2, Coronavirus infections

## Abstract

**Objective:**

To compare the effectiveness and safety of non-mRNA versus mRNA COVID-19 vaccines on pregnant women and their newborns in a systematic review with meta-analysis.

**Data sources:**

We searched PubMed, Embase, and Cochrane Central in May 2023.

**Study selection:**

The search strategy yielded 4451 results, 16 studies were fully reviewed. We selected case-control studies analysing non-mRNA versus mRNA vaccines. Data collection and analysis: we assessed the risk of bias using the Cochrane Risk of Bias in Non-randomized Studies of Interventions (ROBINS-I) tool. Standardised mean differences were pooled using random-effect models.

**Data synthesis:**

We identified 8 prospective and retrospective studies with a total of 32,153 patients. Non-mRNA vaccines were associated with a higher incidence of fever (OR 2.67; 95% CI 2.08-3.43; p<0.001), and a lower incidence of fetal or neonatal death (OR 0.16; 95% CI 0.08-0.33; p<0.001). In subgroup analyses, the Jansen vaccine (Ad26.COV2.S) was found to have a higher rate of premature labor/delivery (OR 4.48; 95% CI 1.45-13.83; p=0.009) and missed/spontaneous abortion (OR 1.90; 95% CI 1.09-3.30; p=0.02), as compared with the Pfizer (BNT162b2) vaccine.

**Conclusion:**

non-mRNA vaccines are associated with a lower incidence of fetal or neonatal death among pregnant women who receive a Covid19 vaccine, although at an increased rate of pyrexia compared with mRNA vaccines. Other studies are required for better assessment.

**PROSPERO:**

CRD42023421814

## Introduction

Pregnant women are a high-risk group for severe Coronavirus 19 (COVID-19) infection, with significant increases in ICU admissions, invasive mechanical ventilation, and mortality rates compared to women of reproductive age who are infected.^(1)^ Recent evidence shows an elevated risk of adverse obstetric outcomes in pregnant women with COVID-19,^(2)^ including preeclampsia, preterm birth, and stillbirth even in asymptomatic patients, which highlights the need for effective prevention measures in this population.^(3)^

In the United States, Pfizer-BioNTech and Moderna (both messenger ribonucleic acid [mRNA] COVID-19 vaccines), and Johnson & Johnson (an adenoviral COVID-19 vaccine) are considered safe for use in pregnant women.^(4)^Other non-mRNA vaccines are approved and widely used in different countries, with evidence of neutralizing antibodies transmission from mother to fetus through the placenta.^(5,6)^ The American College of Obstetricians and Gynecologists strongly recommends vaccination for pregnant women without expressing a preference for any specific approved vaccine in the United States.^(7)^However, the Royal College of Obstetricians and Gynecologists recommends the use of mRNA vaccines due to the more robust data supporting its use.^(8)^

Despite guideline recommendations, adherence to vaccination in pregnant women remains low,^(7)^likely due to concerns about potential long-term implications of vaccination during pregnancy.^(8,9)^ Previous meta-analyses have mainly compared vaccinated to unvaccinated populations,^(10-16)^ and almost exclusively assessed mRNA vaccines.^(10,11,16,17)^ These previous analyses had limited outcome measures, were performed before recent large-scale studies were made available, and assessed limited duration of follow-up. Therefore, there is an unmet need to compare mRNA vs. non-mRNA vaccines for pregnant women.^(17)^

Given recent publications assessing the use of non-mRNA vaccines, we performed a systematic review and meta-analysis comparing the effectiveness and safety of non-mRNA versus mRNA COVID-19 vaccines on pregnant women and their newborns.

## Methods

This systematic review and meta-analysis was performed according to the Cochrane Collaboration and the Preferred Reporting Items for Systematic Reviews and Meta-Analysis (PRISMA) statement recommendations.^(18)^

We systematically searched PubMed, Embase, and Cochrane Central Register of Controlled trials in May 2023. We used the following medical subject heading terms: ‘COVID-19’, ‘vaccine’, and ‘pregnancy’. The complete search strategy can be found in the Supplemental material:chart S1. We restricted inclusion in this meta-analysis to studies that met all the following eligibility criteria: (1) study population composed of pregnant women; (2) head-to-head comparison of mRNA versus non-mRNA vaccines; and (3) clinical studies. There was no time or language restriction. We excluded studies with (1) overlapping patient populations; or (2) no specifications of vaccine type.

No filters or language restrictions were applied in our search. We also utilized a technique of backward snowballing, searching for additional eligible studies through a review of the references from prior publications, including meta-analyses and included studies. Study screening was carried out independently by two authors, following the predefined search criteria. Eventual conflicts were resolved by consensus among the authors.

Two authors extracted outcome data independently and a third author ensured that data was consistent for statistical analysis. From each article the following standard information was extracted: publication year; country, study design, sample size, and characteristics of the participants. Two authors independently extracted baseline characteristics of the study population, including comorbidities. Patient-level data was not requested.

Maternal outcomes of interest were: (1) premature labor; (2) spontaneous abortion; (3) study-defined pregnancy complications; (4) side effects, such as pyrexia, myalgia, fatigue, or low mood. Neonatal outcomes of interest were: (1) neonatal or fetal death; (2) fetal disorders. We performed subgroup analyses according to the type of vaccine, such as: Pfizer (BNT162b2), Moderna (mRNA 1273), Astrazeneca’s (AZD1222), Jansen’s (Ad26.COV2.S) and Sinovac’s (Sinovac-CoronaVac).

Binary outcomes were summarized using the DerSimonian and Laird random effect model, with odds ratios (OR) and 95% confidence intervals (CI) as measures of effect size. Statistical heterogeneity was assessed by I2 and Cochran Q, and heterogeneity was considered significant if p-value < 0.10 and I^2^ > 25%. We performed sensitivity analyses using the leave-one-out strategy as well as Baujat plots. Review Manager 5.1 (Nordic Cochrane Centre, The Cochrane Collaboration, Copenhagen, Denmark) and RStudio (PBC, Boston, MA) were used for statistical analysis and data conversion, if needed.

The quality of studies included was appraised using the Cochrane Risk of Bias in Non-randomized Studies of Interventions (ROBINS-I) tool.^(19)^Two authors completed the risk assessment tool independently, and disagreements were resolved by discussing the discrepancies. Small study effect (publication bias) was assessed with funnel plots for the outcomes of pregnancy complications, fetal disorders, and premature labor/delivery.

## Results

**Figure 1:**
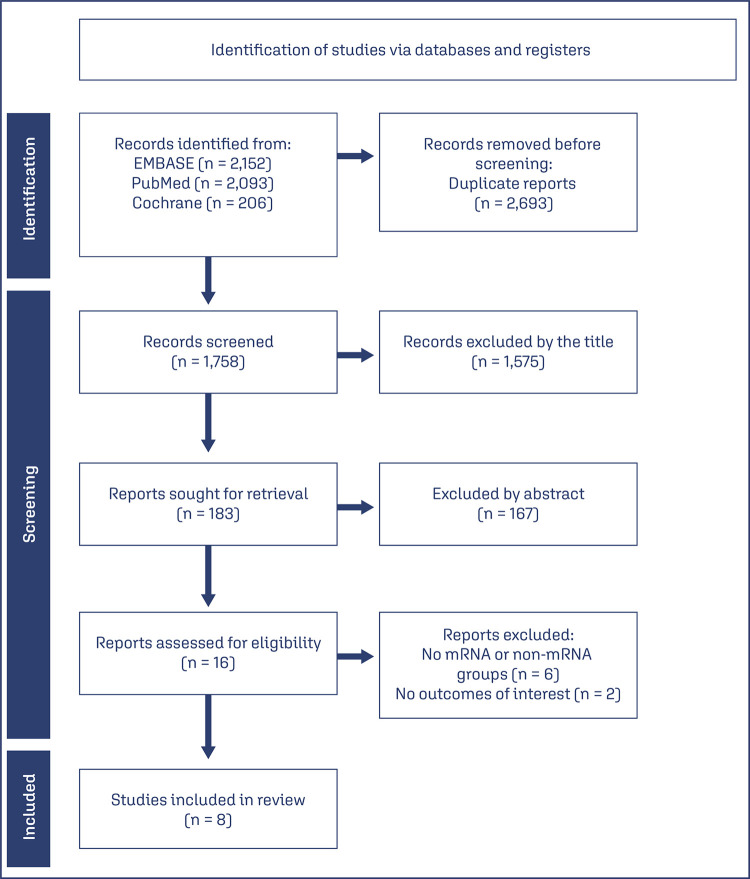
PRISMA 2020 flow diagram for study selection on systematic reviews

The search strategy yielded 4451 results. After removal of duplicate records and relevant exclusions, 16 studies were selected and fully reviewed according to the inclusion criteria (Figure 1). After relevant exclusions, we included eight observational studies, with a total of 32,187 pregnant women, of whom 26,428 (82.1%) received mRNA vaccines and 5,725 (17.89%) received non-mRNA vaccines. Out of the mRNA vaccines, 16,011 (60.5%) were Pfizer’s (BNT162b2), 5,006 (18.9%) were Moderna’s (mRNA 1273) and 5,411 (20.5%) were unspecified. As for non-mRNA, 4,965 (85.9%) were Astrazeneca’s (AZD1222), 106 (1.9%) were Jansen’s (Ad26.COV2.S) and 688 (12.2%) were Sinovac’s (Sinovac-CoronaVac). The baseline characteristics of included studies are reported on chart 1.

**Chart 1:** Baseline characteristics of included studies

Study	Design	Country	Number of patients	Vaccines type	Age	Race			
						White	Black^1^	Other^2^	Unknown
Calvert et al. (2023)^(2)^	Retrospective Cohort Study	Scotland	G1: 1202 G2: 5411	Astrazeneca *versus* Pfizer	31.8 ± 5.1	G1: 1119 G2: 4893	G1: 20 G2: 65	G1: 61 G2: 410	G1: 21 G2: 130
Jacob-Chow et al. (2022)^(20)^	Retrospective Cohort Study	Singapure and Malaysa	G1: 245 G2: 1539	Astrazeneca and Sinovac *versus* Pfizer and Moderna	32.7 ± 3.9	NA	NA	G1: 245 G2: 1539	NA
Kant et al. (2022)^(21)^	Prospective Cohort Study	Netherlands	G1: 4 G2: 45	Astrazeneca and Jansen *versus* Pfizer and Moderna	32.6 ± 3.2	NA	NA	NA	NA
Kobayashi et al. (2022)^(22)^	Retrospective Cohort Study	Brazil	G1: 439 G2: 143	Astrazeneca and Jansen *versus* Pfizer	NA**	G1: 207 G2: 61	G1: 187 G2: 63	G1: 1 G2: 1	G1: 44 G2: 18
Magnus et al. (2022)^(23)^	Retrospective Cohort Study	Sweden and Norway	G1: 264 G2: 15377	Astrazeneca *versus* Pfizer and Moderna	NA**	NA	NA	NA	NA
Mascolo et al. (2022)^(24)^	Retrospective Cohort Study	EudraVigilance	G1: 619 G2: 2612	Astrazeneca and Jansen *versus* Pfizer and Moderna	NA**	NA	NA	NA	NA
Qiao et al. (2021)^(25)^	Retrospective Cohort Study	Brazil	G1: 2545 G2: 788	Astrazeneca, Jansen and Sinovac *versus* Pfizer	NA**	G1: 843 G2: 220	G1: 779 G2: 266	G1: 10 G2: 2	G1: 272 G2: 82
Vuong et al.* (2022)^(26)^	Prospective Cohort Study	Viet Nam	G1: 441 G2: 513	Astrazeneca *versus* Pfizer	30.4 ± 4.5	NA	NA	NA	NA

(*) Correspondence. EudraVigilane - European Union Drug Regulation Authorities Pharmacovigilance; (**) Maternal age was stratified into age groups, giving the amount of women who fitted a certain range, not being possible to calculate the mean value; G1 - Stands for Group 1, which is the intervention (non-mRNA vaccine); G2 - Stands for Group 2, which is the control (mRNA vaccine); ^1^ - Includes Black, Brown, Caribbean, or African ethnicity; ^2^ -“Other” stands for Asian, mixed, or other; NA - Not Available

### Maternal outcomes

**Figure 2:**
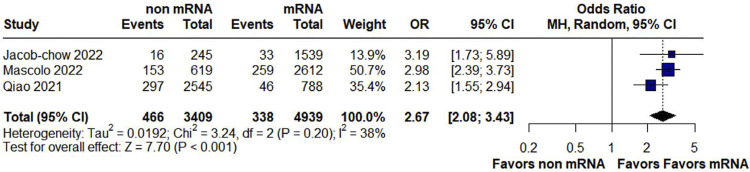
Forest plot for pyrexia (fever) for non-mRNA versus mRNA vaccines

**Figure S1:**
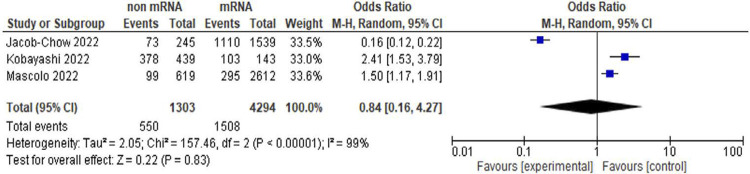
Forest plot of summary of crude ORs and 95% CIs for non-serious adverse events for non-mRNA *versus* mRNA vaccines

**Figure S2:**
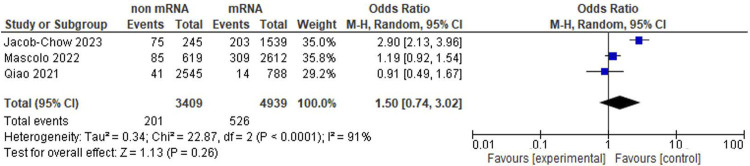
Forest plot of summary of crude ORs and 95% CIs for fatigue and/or low mood for non-mRNA *versus* mRNA vaccines

**Figure S3:**
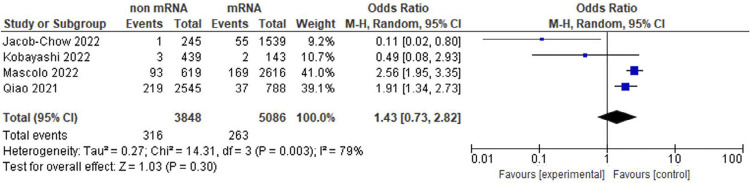
Forest plot of summary of crude ORs and 95% CIs for myalgia and/or soreness for non-mRNA *versus* mRNA vaccines

**Figure S4:**
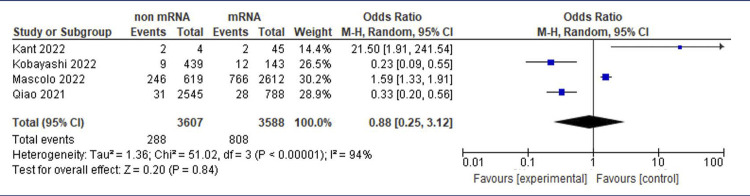
Forest plot of summary of crude ORs and 95% CIs for pregnancy complications for non-mRNA *versus* mRNA vaccines

**Figure S5:**
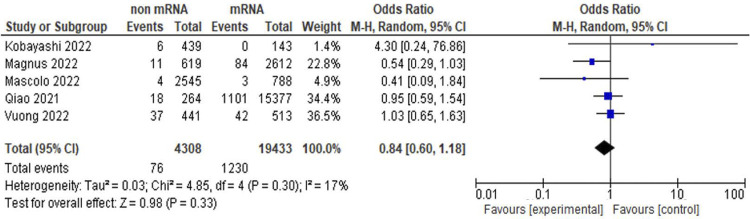
Forest plot for premature labor/delivery for non mRNA *versus* mRNA vaccines

**Figure S6:**
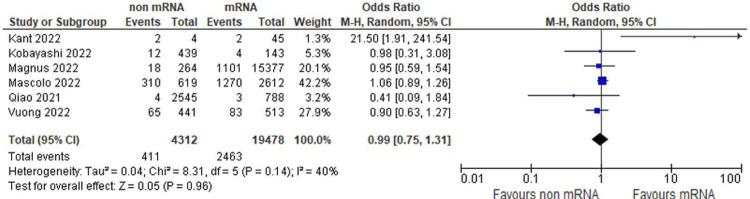
Forest plot for missed/spontaneous abortion for non mRNA *versus* mRNA vaccines

In the pooled analysis of maternal outcomes, non-mRNA vaccines increased the risk of fever compared to mRNA vaccines (OR 2.67, 95% CI 2.08 to 3.43; p <0.001; *I*^2^ = 38%) (Figure 2). The remaining outcomes were not statistically different between mRNA and non-mRNA vaccines: non-serious events (OR 0.84; CI 95% 0.16 to 4.27; p = 0.83; *I*^2^ = 99%) (Supplemental Material: Figure S1), fatigue/low mood (OR 1.50; 95% CI 0.74 to 3.02; p = 0.26; *I*^2^ = 91%) (Suppl. Material: Figure S2), myalgia/soreness (OR 1.43; 95% CI 0.73 to 2.82; p = 0.30; *I*^2^ = 79%) (Suppl. Material: Figure S3), pregnancy complications (OR 0.88; 95% CI 0.25 to 3.12, p = 0.84; *I*^2^ = 94%) (Suppl. Material: Figure S4), premature labor/delivery (OR 0.84; 95% CI 0.60 to 1.18; p = 0.33; I^2^ = 17%) (Suppl. Material: Figure S5), and missed/spontaneous abortion (OR 0.99; 95% CI 0.75 to 1.31; p = 0.96; I^2^ = 40%) (Suppl. Material: Figure S6).

### Fetal outcomes

**Figure 3:**
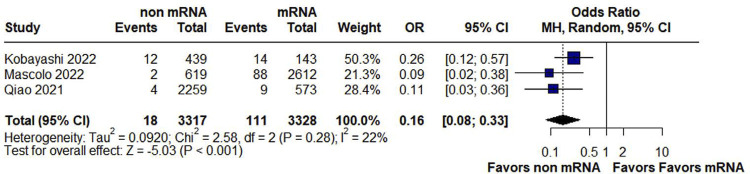
Forest plot of neonatal or fetal death for non-mRNA versus mRNA vaccines

**Figure S7:**
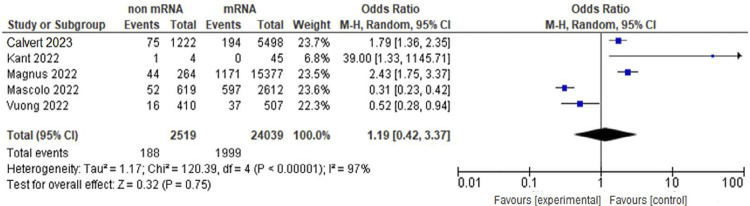
Forest plot of summary of crude ORs and 95% CIs for fetal disorders for non-mRNA *versus* mRNA vaccines

Non-mRNA vaccines were significantly associated with fewer fetal deaths, as compared with mRNA vaccines (OR 0.16; 95% CI 0.08 to 0.33; p <0.001; *I*^2^ = 22%) (Figure 3). There was no statistical difference between non-mRNA and mRNA vaccines in fetal disorders (OR 1.19, 95% CI 0.42 to 3.37; p = 0.75; *I*^2^ = 97% (Suppl. Material: Figure S7).

### Subgroup analyses

**Figure S8:**
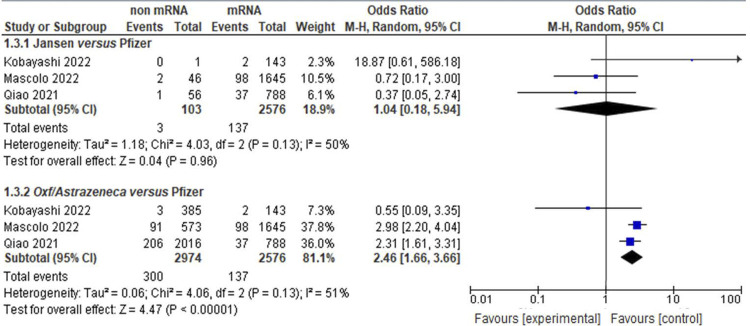
Forest plot of summary of crude ORs and 95% CIs for premature labor/delivery for Jansen and Astrazeneca (non-mRNA vaccines) *versu*s Pfizer (mRNA vaccine)

**Figure S9:**
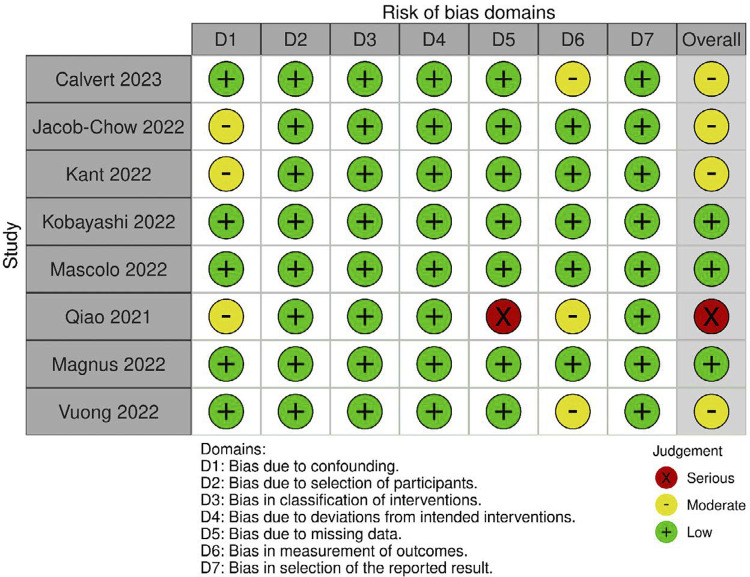
Forest plot of summary of crude ORs and 95% CIs for missed/spontaneous abortion for Jansen and Astrazeneca (non-mRNA vaccines) *versu*s Pfizer (mRNA vaccine)

**Figure S10:**
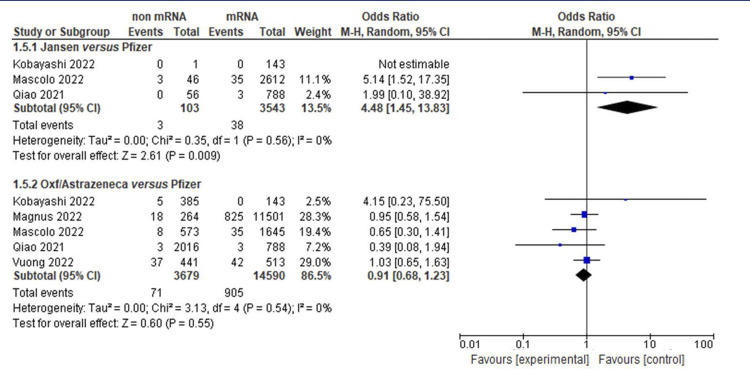
Forest plot of summary of crude ORs and 95% CIs for myalgia or soreness for Jansen and Astrazeneca (non-mRNA vaccines) *versu*s Pfizer (mRNA vaccine)

In subgroup analyses stratified by vaccine type, the Jansen vaccination showed increased the risk of premature labor/delivery (OR 4.48, 95% CI 1.45 to 13.83; p 0.009; *I*^2^ = 0%); (Suppl. Material: Figure S8) and missed/spontaneous abortion (OR 1.90, 95% CI 1.09 to 3.30; p = 0.02; *I*^2^ = 0%); (Suppl. Material: Figure S9) when compared with Pfizer. There were no significant differences between groups in soreness or myalgia (OR 1.04, 95% CI 0.18 to 5.94; p 0.95; I^2^ = 50%); (Suppl. Material: Figure S10). As for the AstraZeneca vaccine increased the risk of myalgia and/or soreness when compared with Pfizer (OR 2.46, 95% CI 1.66 to 3.66; p < 0.001; *I*^2^ = 51%); (Suppl. Material: Figure S10). There were no significant differences between groups for premature labor (OR 0.91, 95% CI 0.68 to 1.23; p = 0.55; *I*^2^ = 0%); (Suppl. Material: Figure S8) or missed/spontaneous abortion (OR 0.80, 95% CI 0.19 to 3.35; p = 0.76; *I*^2^ = 94%); (Suppl. Material: Figure S9).

### Quality assessment

**Figure 4:**
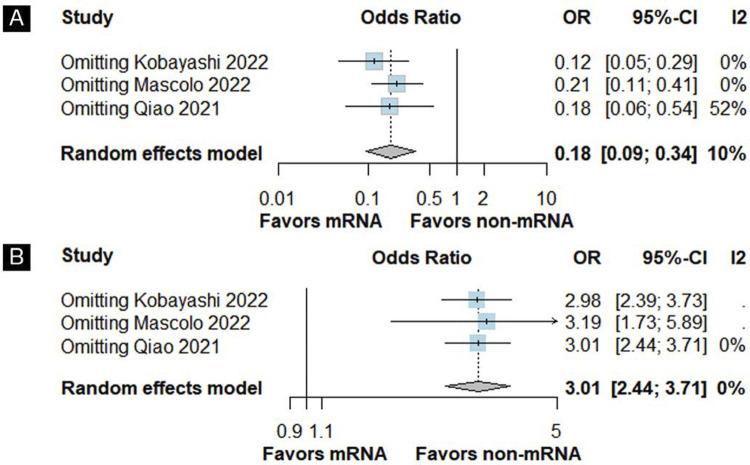
Leave-one-out forest plot for fetal or neonatal death (A) and for pyrexia (B)

**Figure S12:**
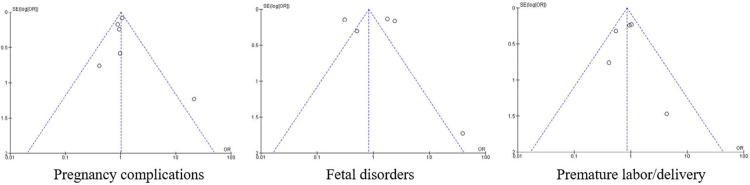
Funnel plot for pregnancy outcomes, fetal disorders and premature labor/delivery

**Figure S13:**
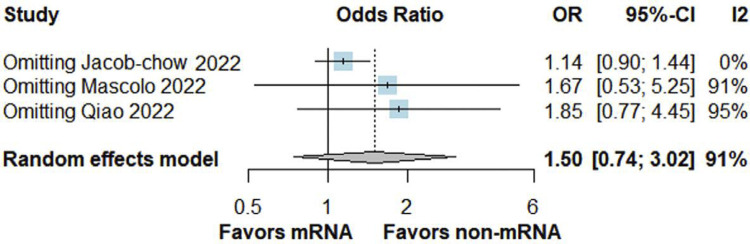
Leave-one-out plot for fatigue or low mood

**Figure S14:**
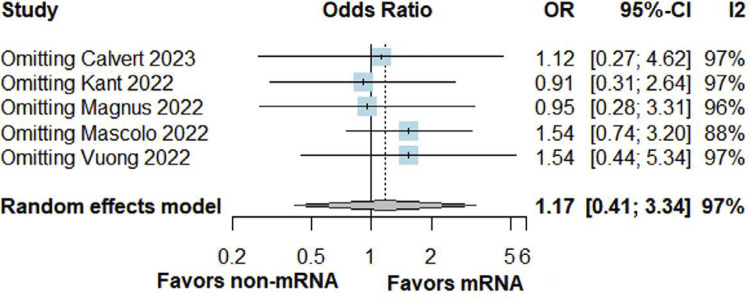
Leave-one-out plot for fetal disorders

**Figure S15:**
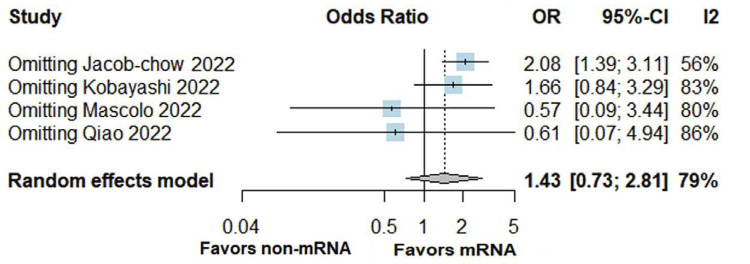
Leave-one-out plot for myalgia or soreness

**Figure S16:**
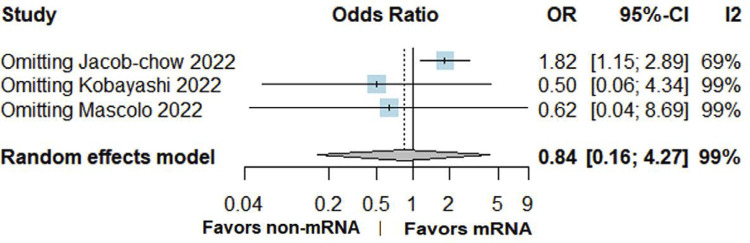
Leave-one-out plot for non serious adverse events

**Figure S17:**
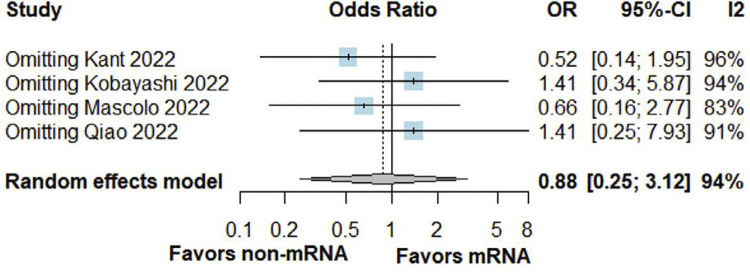
Leave-one-out plot for pregnancy complications

**Figure S18:**
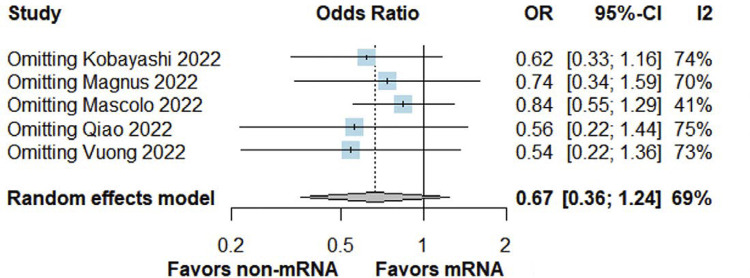
Leave-one-out plot for premature labor

**Figure S19:**
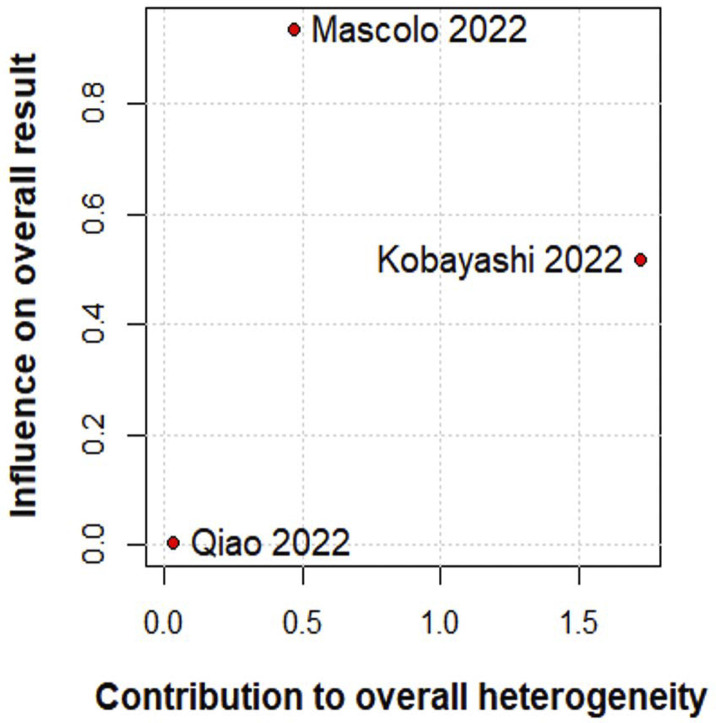
Baujat plot for fetal or neonatal death

**Figure S20:**
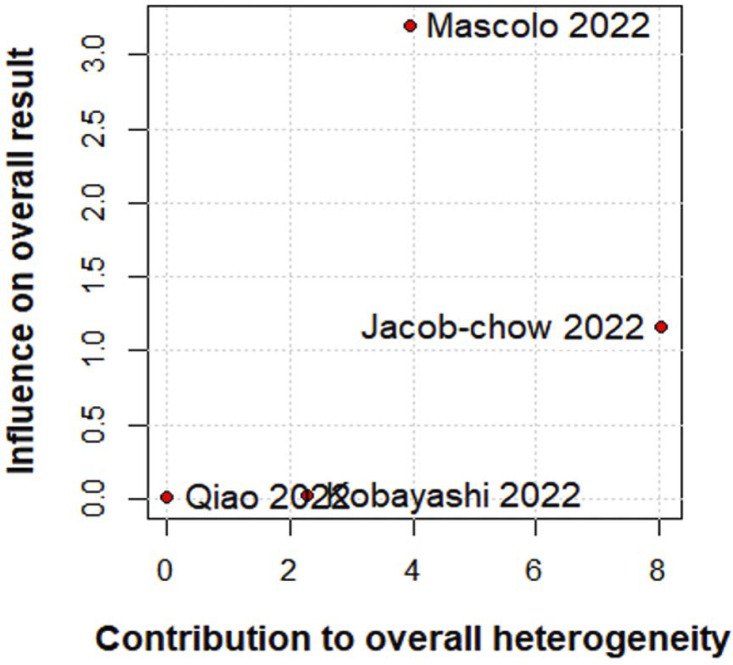
Baujat plot for myalgia or soreness

**Figure S21:**
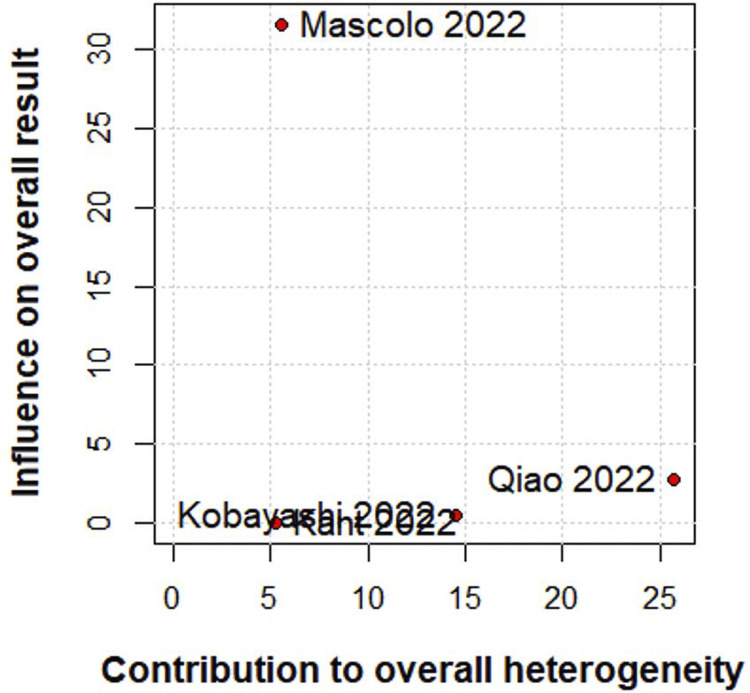
Baujat plot for pregnancy complications

**Figure S22:**
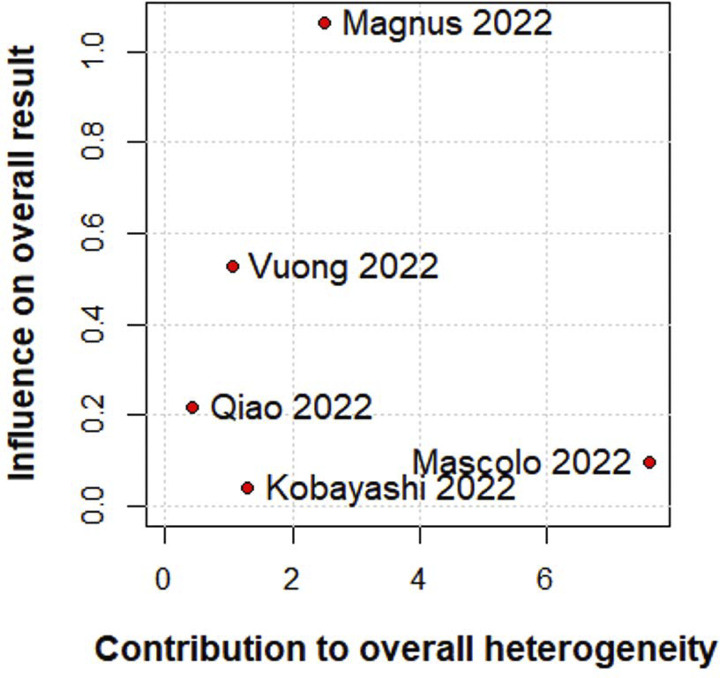
Baujat plot for premature labor

**Figure S23:**
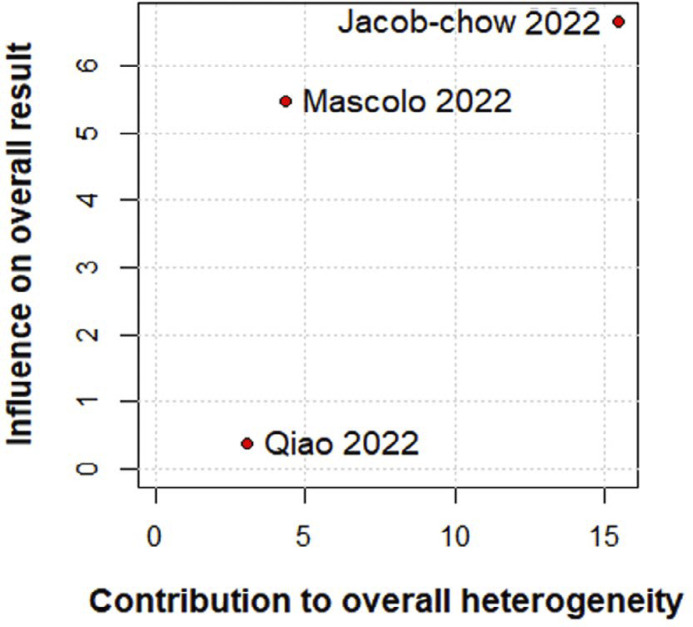
Baujat plot for fatigue or low mood

**Figure S24:**
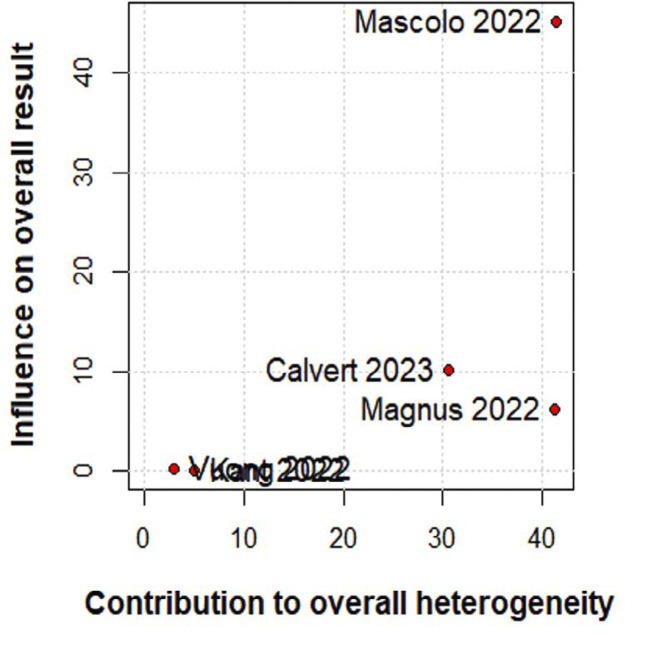
Baujat plot for fetal disorders

**Figure S25:**
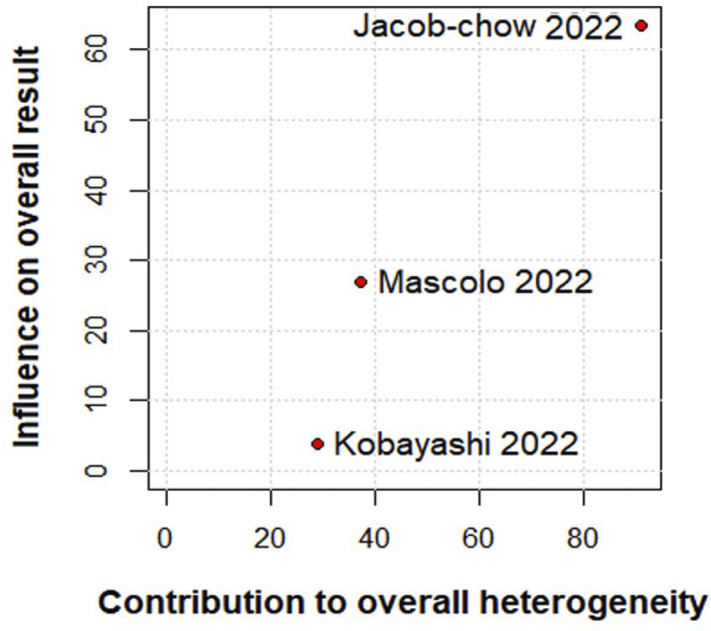
Baujat plot for non-serious adverse events

The risk of bias assessment of each study is provided in the suppl. Material figure S9. Four studies were classified as moderate risk of bias, two due to confounding^(20,21,25)^and two in the measurement of outcomes domain.^(2,25,26)^One was classified as “serious risk of bias” due to missing data,^(25)^and the remaining studies were assessed as low risk of bias. Funnel plots for pregnancy complications outcome showed some evidence of publication bias due to asymmetric distribution of study weights around the pooled study results (Suppl. Material: Figure S12). Unfortunately, Egger’s regression test could not be performed to evaluate and confirm potential publication bias due to the limited number of included studies (n < 10). We explored the consistency of treatment effects using the leave-one-out strategy (Figure 4) ( Figure S13-18) and Baujat plots (Suppl. Material: Figure S19-25), which revealed that Jacob-Chow et al. (2022)^(20)^and Mascolo et al. (2022)^(24)^ were mainly responsible for driving the high heterogeneity observed, as confirmed by the Baujat plots. Yet, results remained consistent with the overall analyses for fetal or neonatal death and pyrexia even when each individual study was removed from the pooled result (leave-one-out sensitivity analysis).

## Discussion

In this systematic review and meta-analysis of eight studies with a total of 32,153 pregnant women, we assessed the effectiveness and safety of non-mRNA compared with mRNA COVID-19 vaccines for maternal and neonatal outcomes. Our main findings were as follows: 1) non-mRNA vaccines were associated with a lower incidence of fetal or neonatal death; 2) non-mRNA vaccines were associated with a higher incidence of fever; and 3) the Jansen non-mRNA vaccine was associated with an increase in premature labor/delivery and missed/spontaneous abortion when compared with the Pfizer vaccine.

Recent literature has consistently demonstrated the remarkable efficacy of vaccines against COVID-19 during pregnancy.^(27)^ Among infected pregnant women, those who were vaccinated had fewer ICU admissions, invasive mechanical ventilation, and mortality rates compared with non-vaccinated women.^(27)^ Moreover, the approved vaccines for pregnant women show minimal adverse events, and offer an additional benefit of transferring antibodies to the fetus, thus providing protection against the virus during the early months of life.^(27)^Nevertheless, there are limited data comparing the vaccines and their subtypes in pregnant women.

A recent meta-analysis^(28)^reported stratified results based on vaccine type, evaluating mRNA (BNT162b2 or mRNA-1273) versus adenovirus vaccines (AZD1222 or Ad26.COV2.S). They reported similar findings for hospitalizations and mortality when comparing non-mRNA and mRNA vaccines. However, they highlighted that baseline data for adenovirus vaccines were often missing, which can make it difficult to acquire data properly and fairly. Similar hospitalizations and mortality rates were found among the subgroups, but adenovirus vaccines were less effective in preventing infections when compared with mRNA vaccines. Nonetheless, there were few studies involving non-mRNA vaccines and making a head-to-head comparison of vaccines types,^(28)^especially in pregnant women.^(22)^

To the best of our knowledge, this is the first meta-analysis comparing the safety profile of mRNA and non-mRNA vaccines that were approved for use during pregnancy. Our findings indicate that non-mRNA vaccines were associated with pyrexia compared with non-mRNA vaccines group. This is particularly important since pyrexia during pregnancy poses risks to both the mother and the fetus.^(29)^During early stages of pregnancy, pyrexia can be particularly hazardous, as it may coincide with critical periods of fetal formation.^(29)^

In our subgroup analyses we found a significantly higher incidence of myalgia/soreness in women who received the AstraZeneca vaccine compared with the Pfizer. Additionally, women who received the Jansen vaccine showed a higher incidence of premature labor/delivery and of missed/spontaneous abortions compared with those who received the Pfizer vaccine. It is unclear whether the lower likelihood of premature labor/delivery is potentially linked to receiving the vaccine later in the third trimester for the Pfizer vaccine. This could not be assessed in our meta-analysis, as only two studies provided detailed information about the timing of vaccine administration.^(2,30)^Moreover, the lower incidence of spontaneous abortions in the Pfizer vaccine group may be of particular interest in women with prior miscarriages. These subgroup analyses are explorative and warrant investigation in future clinical trials.

This study has important limitations. First, the eight studies utilized in this analysis were not randomized, potentially introducing confounding bias. Nevertheless, there are significant challenges of conducting randomized controlled trials involving COVID-19 vaccines in pregnant women. Additionally, there is a suggestion that the time interval between vaccination and delivery may affect neonatal antibody titers. Further investigation is warranted to elucidate the impact of timing of vaccine on perinatal outcomes. Lastly, some of our analyses had moderate heterogeneity and must be interpreted with caution. The observed heterogeneity could possibly be attributed to methodological differences between the studies or differences in the vaccine manufacturer. Nevertheless, results were consistent on sensitivity analyses removing one study at a time and recalculating maternal and neonatal outcomes.

## Conclusion

Our findings indicate that non-mRNA vaccines are associated with a lower incidence of fetal or neonatal death among pregnant women who receive a Covid19 vaccine, although at an increased rate of pyrexia compared with mRNA vaccines. These findings may serve as an important aid in the decision-making regarding recommendations of vaccinations to the pregnant women population.

## Figures and Tables

**Figure S11:**
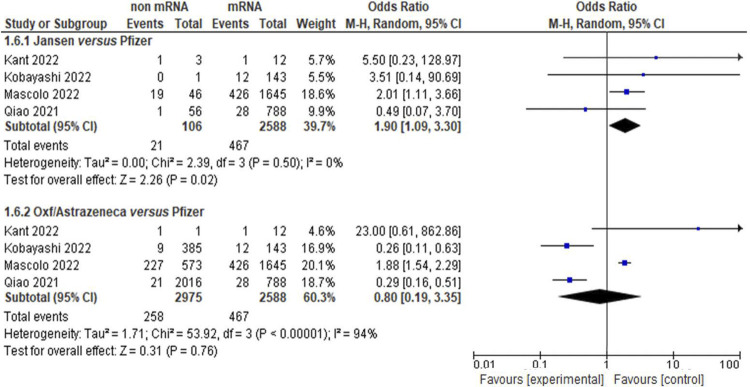
ROBINS-I

## Supplemental material

**Chart S1:** Search strategies used for databases searched

Database	Search Strategy
PubMed	(“COVID-19*” OR “COVID 19*” OR COVID19* OR COVID-19 [mh] OR “2019-nCoV*” OR “2019 nCoV*” OR SARS* OR Coronavirus*) AND (vaccine OR vaccines OR immunization OR immunizations) AND (pregnancy OR pregnancies OR gestation)
Embase	(“COVID-19*” OR “COVID 19*” OR “COVID19* OR COVID-19 OR “2019-nCoV*” OR “2019 nCoV*” OR SARS* OR Coronavirus*) AND (vaccine OR vaccines OR immunization OR immunizations) AND (pregnancy OR pregnancies OR gestation)
Cochrane Central	(“COVID-19*” OR “COVID 19*” OR “COVID19* OR COVID-19 OR “2019-nCoV*” OR “2019 nCoV*” OR SARS* OR Coronavirus*) AND (vaccine OR vaccines OR immunization OR immunizations) AND (pregnancy OR pregnancies OR gestation)
